# The Influence of Printing Speed and Temperature on the Mechanical, Absorptive, and Morphological Properties of PLA-Based Hybrid Materials Produced with an FDM-Type 3D Printer

**DOI:** 10.3390/polym16192771

**Published:** 2024-09-30

**Authors:** Rumeysa İncesu, Tarkan Akderya

**Affiliations:** 1 Department of Biomedical Engineering, Graduate Education Institute, University of Bakırçay, Menemen 35665, Izmir, Turkey; rumeysaincesuu@gmail.com; 2 Department of Biomedical Engineering, Faculty of Engineering and Architecture, University of Bakırçay, Menemen 35665, Izmir, Turkey

**Keywords:** fused deposition modelling, hybridisation, poly (lactic acid), mechanical properties, absorptive properties

## Abstract

Composite materials are used in many engineering applications and industrial fields due to their superior properties, such as high strength, lightweight, and stiffness. These outstanding properties have made these materials an alternative to metallic materials. The vital need for new lightweight and inexpensive materials with superior strength properties has led to research on “hybridisation”. Hybrid composites with more than one type of polymer in the same structure are needed to achieve a better balance of properties and to combine many desired properties in a single material. Many researchers have studied the hybrid effect and contributed to the understanding and modelling of the subject. Studies to explain the primary mechanism of the hybrid effect are limited and insufficient to explain the complex interaction. In this study, a three-dimensional printer using fused deposition modelling technique was used to produce hybrid materials, and the influence of printing parameters on the mechanical, absorptive, and morphological properties of poly (lactic acid) (PLA), Tough PLA, and PLA/Tough PLA hybrid materials were investigated. The hybrid material form exhibited superior properties when selecting specific production parameters from individual raw elements. It can be said that the mechanical properties of the PLA/Tough PLA hybrid material increased with the increase in production temperature.

## 1. Introduction

In recent years, composites have reached an increasing usage trend due to their many superior properties, such as high strength values, ultra-light weight, high thermal capabilities, and anti-corrosive structure. Thanks to these properties, they are being used in transportation, defence, aircraft, space, and biomedical fields. With the increasing use of composite materials reinforced with synthetic fibres, the accumulation of synthetic fibres in the earth and their negative contributions to sustainability have come to the fore. In this context, research on biocomposite materials that can be an alternative to composite mate-rials with artificial fibres has started. Studies and research are ongoing to determine the characteristic properties of biopolymers with high rigidity, mechanical properties, thermal stability, and anti-corrosive structure thanks to the fibres that can be obtained from nature and used in three-dimensional (3D) printer technology.

Although obtaining structures that can be produced with fused deposition modelling (FDM) technology by using multiple polymers excites researchers, it has not been found to be a serious research area. Due to the newness of the technology and the wide variety of production parameters, parametric studies on this production area and their effects on the characteristic properties need to be determined.

With the development of the FDM production technique, the focus has been on mate-rials that can be used in FDM-type 3D printers. The pure polymer filament material used in this type of printer, which generally has a low melting point and low viscosity, flows easily from the nozzle tip during printing, allowing for the layers to deposit and adhere to each other. Thermoplastics have become the most widely used material in FDM techniques due to their ease of processing, low cost, and low moisture absorption [1,2]. The nozzle diameter used in an FDM printer can have dimensions ranging from 0.1 to 1 mm, and the operating temperature ranges from 150 to 300 °C. The diameter of the filament used as consumable material ranges from 1.75 to 3 mm, and today, an FDM filament with a diameter of 1.75 mm is considered standard [1,3].

**Figure 1:**
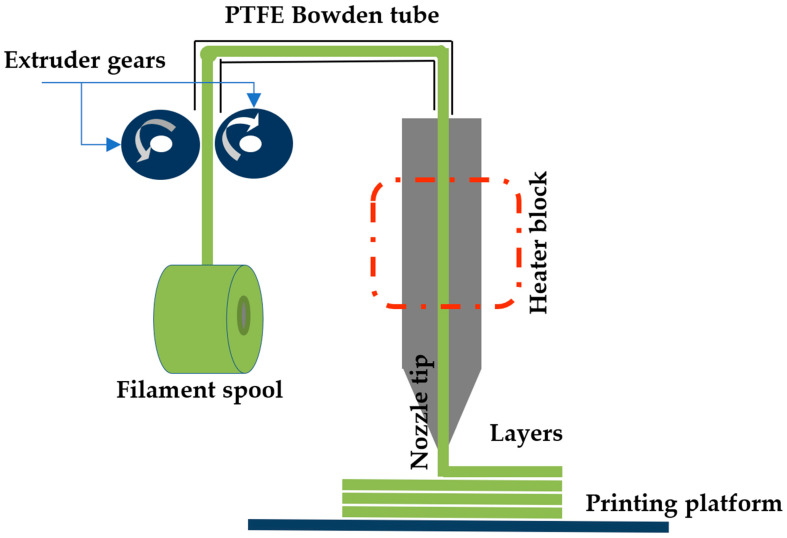
Typical FDM-type 3D printer system schematic.

The thermoplastic polymer filament material, which is melted through a singularly heated nozzle, passes between two gears moving in opposite directions, and the filament is brought to the desired temperature and softened by the temperature control mechanism. The desired model is obtained by extruding the molten polymer material from the nozzle (hotend) in accordance with the G-code instructions and depositing the layers on the printing table by stacking them on top of each other. After the first layer is printed, the printing table moves downward in the direction of the z-axis in the thickness of one layer so that the subsequent layer merges with the printed layer and the model is produced layer by layer [4]. Figure 1 presents a schematic view of a typical FDM-type 3D printing technique.

**Figure 2:**
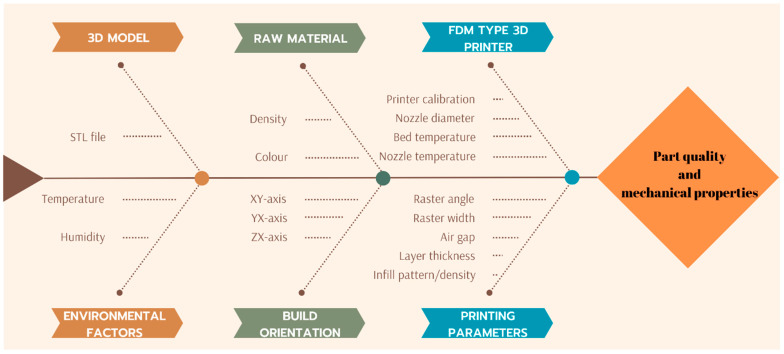
Cause-and-effect relationship of the general process parameters of the FDM printer.

FDM, a rapid manufacturing technique capable of producing complex parts layer by layer in less time than traditional manufacturing methods, offers a challenging process for part production despite its many advantages. In order for the 3D part produced with FDM technology to meet certain standards and specifications, the printing parameters that affect the quality, efficiency, and mechanical properties of the final product must be carefully selected. Nozzle temperature, nozzle diameter, bed temperature, raster angle, raster width, layer thickness, infill pattern, and infill density are among the basic parameters used. In addition, these parameters are usually set through slicing software after design, before data are entered into the FDM device [1,5]. In Figure 2, the cause-and-effect relationship of the general process parameters of the FDM printer is shown in a diagram.

In a study conducted by He and Khan [6], acrylonitrile butadiene styrene (ABS) specimens with larger nozzle diameter and higher layer thickness showed a higher fatigue resistance. Accordingly, the micro-void area decreased along with the number of micro-voids per unit area. Vinitha et al. [7], who studied the effect of printing speed on the surface quality of specimens produced by FDM-type printer, found that decreasing the printing speed improves the surface quality of the product, and the effect of printing speed can be neglected when printing thinner layers. Geng et al. [8], who investigated the effect of extrusion speed and printing speed on the dimensional stability of PEEK-based parts, concluded that printing speed that does not work in harmony with extrusion speed produces a specimen with disproportionate dimensions. Caminero et al. [9], who investigated the impact property effect of Kevlar, glass, and continuous fibre-reinforced polyamide (PA) composites with layer thickness, structure orientation, and fibre volume variation, found that layer thickness has a higher effect on the strength of the printed parts. Ayrılmış et al. [10], who examined the effect of layer thickness on the water absorbency and mechanical properties of wood/PLA composite material, observed that the porosity of the printed specimen increases with increasing layer thickness, and the voids filled with water due to the pores form. Thus, they concluded that as the layer thickness increases, water absorption also increases. Rahman [11] investigated the mechanical behaviour of the specimen printed with PEEK material by FDM at different raster angles and observed that the highest strength value was obtained at the 0° scan angle. Fatimatuzahraa et al. [12], who investigated the mechanical properties of ABS specimens printed with FDM with different raster angles, concluded that the specimen produced with linear diagonal lines with 45°/−45° angles has the highest strength in flexural and impact properties. Akderya [13] investigated the mechanical, absorptive, and morphological properties of PLA specimens produced with an FDM-type 3D printer after ultraviolet post-curing. In the study, the effect of post-production processes on the characteristic properties of PLA specimens was revealed, and the effect of printing temperature was also investigated. The study revealed that increasing the printing temperature causes the material to have better flexural properties.

Accordingly, the main objective of this research is to fabricate hybrid material specimens made of two different polymer materials with different properties by additive manufacturing method and then to determine their mechanical, absorptive, and morphological properties to obtain information for the characterisation of these specimens. Hybridisation was achieved by using more than one material in the same layer and even in the same printing fibre with the printer produced. Thus, different materials can be used in different ratios in the same layer, and functionally graded material production can be carried out. An intermediate form material can be obtained with materials with good interfacial bonding ability in the same printing fibre. An FDM-type 3D printer that can produce specimens with hybridisation or functional grading processes was developed in this context. Hybridised materials provide higher performance by eliminating the damages caused by interlayer forces, and based on this, they find application areas in industries such as the aerospace industry.

## 2. Materials, Methods, and Specimen Preparation

### 2.1. FDM-Type 3D Printer Revision

**Figure 3:**
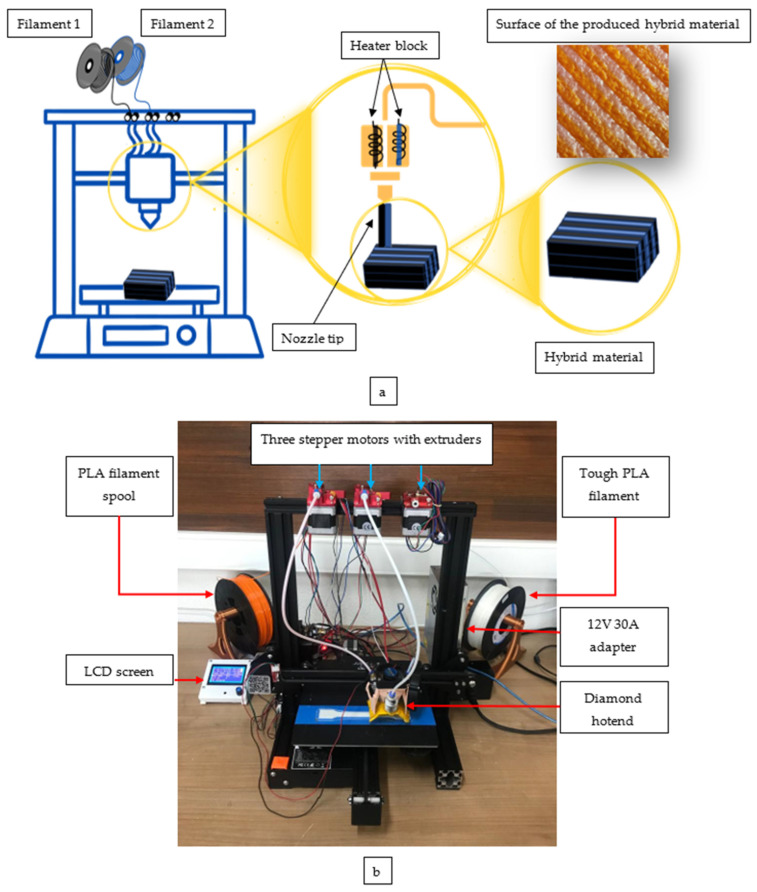
(**a**) Schematic view of the hybrid-type 3D printer; (**b**) FDM-type 3D printer capable of hybrid specimen production.

In the first stage, an FDM-type printer was revised for use in this study. The most important factor in the printer being revised in terms of hardware and software and being able to produce hybrid materials is that the raw materials that make up the hybrid material can be adjusted in volumetric terms at the desired rates during hybrid production. The ability to try various filaments with good interlayer and printing line bonding ability provides freedom in the research and development of new hybrid forms. In addition, performing hybridisation simultaneously can provide a more controlled production environment for observing the hybrid effect affecting the material characteristics. In order to revise the FDM-type 3D printer, the old extruder and hotend/nozzle set were decommissioned and replaced with a diamond hotend to enable the FDM-type 3D printer to produce with multiple filaments simultaneously. The design part of the diamond hotend was produced redundantly, where different nozzle outputs are fixed and allowed for mixing by feeding filament from three different channels and melting polymer filaments in a crucible. In the first stage of this study, two nozzles were actively operated, and hybrid mixing of two different materials was achieved. The flat motor holder apparatus was designed and manufactured to attach the stepper motors to the upper part of the printer frame and successfully send the filament to the diamond hotend using the extruder gears. By means of 6-channel sigma profile nuts, the flat motor holder apparatus was mounted on the sigma profile in a non-slip and vibration-free manner. For the extruder stepper motor and nozzles to communicate with the motherboard healthily, cables were arranged with 1-pin and 4-pin female connectors. In order for the commands from the motherboard to reach the parts, the ends of the cables must be arranged with connectors in this way in terms of longevity and transmission health. Ramps (RepRap Arduino Mega Pololu Shield) 1.4 is used with the Arduino IDE 2.3.3 software board Mega 2560 (Smart Projects Co., Ltd., Pescara, Italy), a control board. Ramps 1.4 can be used with the Arduino Mega model or many cards with similar pin sequences, thanks to its shield structure. It can also be used by sitting directly on the Arduino Mega and allows for the control of 5-step motors. Appropriate stepper motors can be controlled by installing 5 A4988 motor driver cards on the product. Ramps supports one motor for the x-axis, one motor for the y-axis, two motors for the z-axis, and two stepper motors for the extruder. In addition to these features, there are also three heater outputs and three limit switch inputs. In addition, the LCD screen input is also compatible with Ramps, provided by the connector on the product. A 12 V 30 A adapter was provided for the power supply of the entire system. In this way, after the printer revision was provided, the first step was taken to make the desired hardware and software changes. Thus, became possible for the nozzle feeds and hybrid material production with multiple extruders to work in harmony. Figure 3 shows the revised FDM-type 3D printer capable of hybrid production.

### 2.2. Specimen Preparation

In order to examine the effect of the production parameters on the mechanical properties of the specimens, trial productions were carried out on the revised printer. After it was concluded that there were no problems in the hybrid production process, it was checked that the dimensional accuracy and properties of the produced specimens were within the tolerance limits, and hybrid absorption, tensile, and three-point bending specimen productions were carried out with the desired production parameters. The volumetric hybridisation ratio was determined as 50%. PLA/Tough PLA hybrid material was obtained by using 50% PLA and 50% Tough PLA volumetrically.

**Figure 4:**
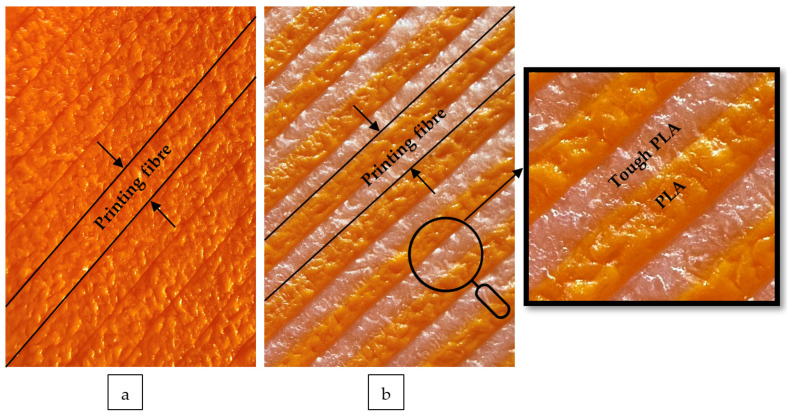
(**a**) Outermost layer view of the PLA specimen; (**b**) outermost layer view of the PLA/Tough PLA hybrid material.

In this study, pure PLA with a density of 1248 kg/m^3^, a melt volume rate of 21.2 cm^3^/10 (220 °C, 5 kg) min, and a melting temperature of 151 °C and Tough PLA material with a density of 1250 kg/m^3^, a melt volume rate of 18.2 cm^3^/10 min (210 °C, 2.16 kg), and a melting temperature in the range of 170–180 °C were used. Pure PLA and Tough PLA, which are non-toxic, non-immunogenic, non-inflammatory, biocompatible, and biodegradable [14], preferred for obtaining the hybrid material, were supplied from BASF (BASF 3D, Emmen, The Netherlands) [15,16]. Tough PLA material is a versatile, industrially used, high-strength filament preferred for printing at high speeds and ideal for printing specimens with unrivalled surface finish [16]. Outermost layer views of the PLA and PLA/Tough PLA hybrid material are given in the Figure 4.

The production temperature and speed were selected as variables based on the production parameters recommended by the manufacturer and used in different studies. Alsoufi et al. [17] investigated the effects of production temperature on the warping deformation, dimensional accuracy, and density of PLA and Tough PLA materials, and they selected the production temperature range from 195 to 250 °C. Wang et al. [18] focused on the effects of the production parameters of an FDM-type printer on the tensile and dynamic mechanical properties of PLA. In the study, the production temperature was selected between 195 and 230 °C. Hsueh et al. [19] studied the effects of printing temperature and speed on the characteristic properties of PLA and polyethylene terephthalate glycol (PETG) materials, and they selected a printing temperature range of 180–220 °C and a printing speed range of 35–45 mm/s for PLA. The printing parameters are given in Table 1.

### 2.3. Characterisation

#### 2.3.1. Mechanical Properties

PLA/Tough PLA hybrid, PLA, and Tough PLA material tensile test specimens were produced on the FDM-type 3D printer in accordance with the ISO 527 standard [20]. Then, all tensile specimens produced at the specified parameters were subjected to tensile tests, and their mechanical properties were analysed. Tensile tests were performed with a Shimadzu brand (Shimadzu Co., Ltd., Kyoto, Japan) universal testing machine with a maximum load capacity of 100 kN, equipped with a load cell and a video extensometer that can measure the amount of elongation. In this device, which enables the determination of the mechanical properties of the material, the elongation and force values of the material were measured by applying unidirectional force to the material with a tensile speed of 1 mm/min.

**Figure 5:**
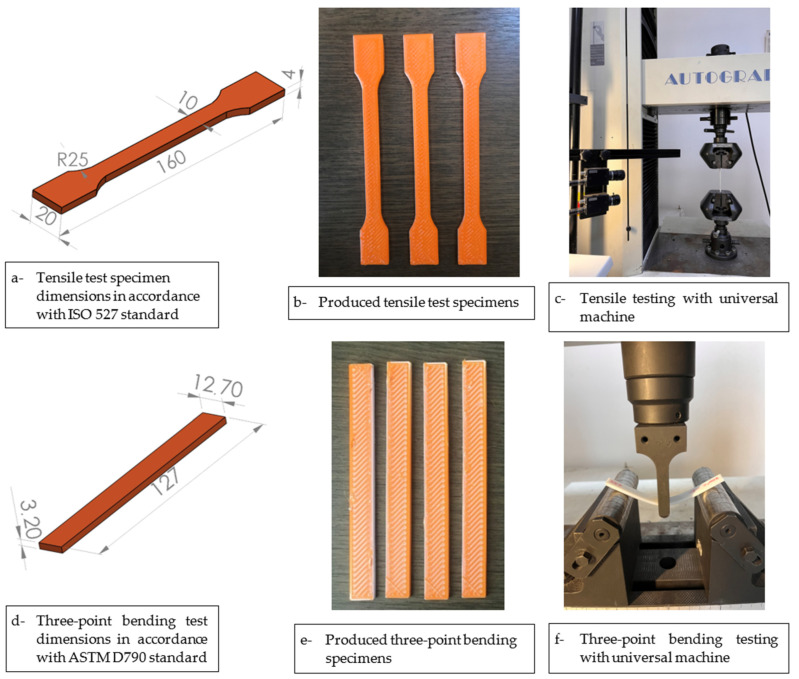
(**a**) Tensile test specimen dimensions in accordance with ISO 527 standard; (**b**) produced tensile test specimens; (**c**) tensile testing with universal machine; (**d**) three-point bending test dimensions in accordance with ASTM D790 standard; (**e**) produced three-point bending specimens; (**f**) three-point bending testing with universal machine.

PLA/Tough PLA hybrid material, PLA, and Tough PLA three-point bending test specimens were produced on the FDM-type 3D printer in accordance with the ASTM D790 standard [21]. A three-point bending test apparatus was attached to the Shimadzu 100 kN tensile testing machine. The flexural properties of the specimens were determined with a test speed of 1 mm/min. The PLA/Tough PLA hybrid material tensile and three-point bending test specimen dimensions, produced test specimens, and photographs taken during the tests are shown in Figure 5.

Five specimens were printed for each parameter. The obtained data are given by taking the arithmetic average of all specimens. In addition, the standard deviation was calculated by taking all samples into account. The formula used to calculate the standard deviation is given below as Equation (1):

(1) $$\sigma =\sqrt{\frac{\sum_{i=1}^{n}{\left(x_{i}-\bar{x}\right)}^{2}}{n}}$$

where σ is the standard deviation, $\bar{x}$ is the sample mean, and n is the number of specimens.

#### 2.3.2. Absorptive Properties

PLA/Tough PLA hybrid material, PLA, and Tough PLA and absorption test specimens were produced in accordance with the ASTM D570 standard [22]. Initial weight measurements were performed with a Radwag AS 220/C/2 (Radwag Balances and Scales Co. Ltd., Radom, Poland) analytical balance with an accuracy of 0.0001 g. Then, five specimens of each parameter were exposed to distilled water in a dark environment for three days, one week, and two weeks. The weight change was calculated using Equation (2):

(2) $$\mathrm{Increase}\;\mathrm{in}\;\mathrm{weight},\;\%=\frac{\mathrm{wet}\;\mathrm{weight}\;-\;\mathrm{conditioned}\;\mathrm{weight}}{\mathrm{conditioned}\;\mathrm{weight}}\times 100$$

#### 2.3.3. Morphological Properties

The morphological properties of the PLA/Tough PLA hybrid samples were analysed using SEM analysis. In order to obtain surface micrographs, a Carl Zeiss 300VP (Carl Zeiss Co., Ltd., Oberkochen, Germany) field emission scanning electron microscope with 15 kV acceleration voltage was used in accordance with the ASTM E986 standard [23]. Before examining the surface morphology, the specimens were coated with 5 nm gold vanadium. The plating process was carried out in 120 s under a vacuum with the ION COATER COX EM (COXEM Co., Ltd., Daejeon, Republic of Korea) brand gold plating device.

## 3. Results and Discussion

### 3.1. Tensile Tests

**Figure 6:**
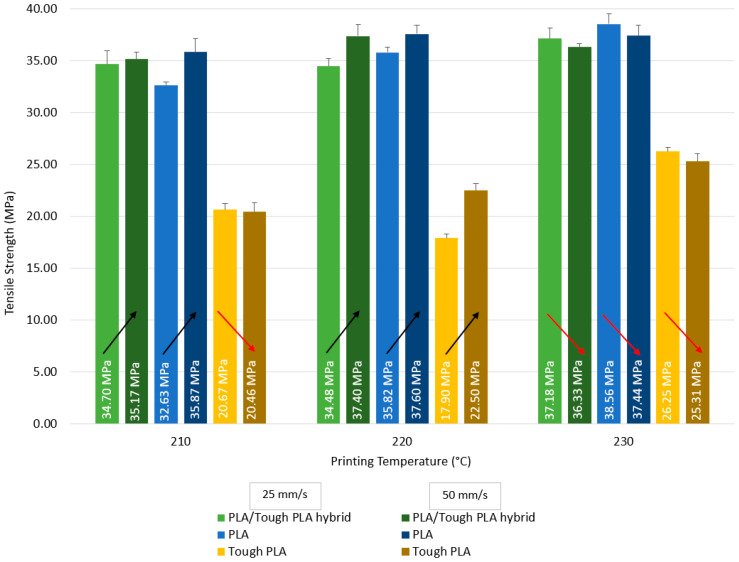
Tensile strength values comparison of the PLA/Tough PLA hybrid, PLA, and Tough PLA specimens. (Arrows indicate the increase and decrease in the tensile strength values of the relevant materials by comparison).

Tensile test results of PLA/Tough PLA hybrid material, PLA, and Tough PLA specimens are given in Figure 6 and Table 2. According to Figure 6, the hybrid specimens produced at 210 and 220 °C have the highest tensile strength value for the ones produced at 50 mm/s printing speed, while the ones produced at 230 °C have the highest tensile strength value when produced at 25 mm/s printing speed. When evaluated in terms of all production parameters, it is seen that the highest tensile strength value for the hybrid materials is 37.40 MPa for the specimens produced at 220 °C with 50 mm/s, and the lowest tensile strength value is 34.48 MPa for the specimens produced at 220 °C with 25 mm/s. When examining printing temperature, there is no visible increase in the specimens printed at 210 and 220 °C with 25 mm/s, while an increase in the tensile strength value is observed at 230 °C. When an evaluation is made in terms of printing speed, it is found that the specimens produced with 25 mm/s have the highest tensile strength value at 230 °C, and the specimens produced at a speed of 50 mm/s have the highest tensile strength value at 220 °C. In addition, it is observed that the specimens produced at 220 °C with 50 mm/s have the highest tensile modulus values (Table 2), which is in line with the tensile strength results.

When Figure 6 is evaluated for the PLA specimens, the specimens produced at 210 and 220 °C have the highest tensile strength value for the specimens produced with 50 mm/s printing speed, while the specimens produced at 230 °C have the highest tensile strength value for the specimens produced with 25 mm/s printing speed. In terms of all production conditions, the highest tensile strength value is 38.56 MPa for the specimens produced at 230 °C with a printing speed of 25 mm/s, and the lowest tensile strength value is 32.63 MPa for the specimens produced at 210 °C with a printing speed of 25 mm/s. The tensile strength value of the specimens produced with 25 mm/s increases as the printing temperature increases. The specimens produced with 25 mm/s printing speed reach the highest tensile strength at 230 °C, while the highest value at 50 mm/s printing speed is reached in the specimens produced at 220 °C. It is revealed that the specimens produced at 210 °C have the highest tensile modulus.

According to Figure 6, the Tough PLA specimens produced at 210 and 230 °C have the highest tensile strength value for the specimens produced with 25 mm/s printing speed, while the specimens produced at 220 °C have the highest tensile strength value for the specimens produced with 50 mm/s printing speed. In terms of all production conditions, the highest tensile strength value is 26.25 MPa for the specimens produced at 230 °C with a printing speed of 25 mm/s, and the lowest tensile strength value is 17.90 MPa for the specimens produced at 220 °C with a printing speed of 25 mm/s. The tensile strength value of the specimens produced with 25 mm/s printing speed decreases after 210 °C, then increases and reaches its highest value at 230 °C. The tensile strength of the specimens produced with 50 mm/s printing speed shows an increasing trend as the printing temperature increases. The highest tensile strength value is reached at 230 °C in the specimens produced using 25 mm/s and 50 mm/s printing speed. It is found that the specimens produced at 230 °C have the highest tensile modulus values.

It is observed that PLA specimens produced at 230 °C have the highest tensile strength for 25 mm/s printing speed. PLA/Tough PLA hybrid and PLA specimens produced at 220 °C with 50 mm/s printing speed, and Tough PLA specimens at 230 °C have the highest tensile strength. When the optimum production conditions were evaluated in terms of the production parameters taken as basis in this study, PLA specimens reach the highest value in terms of tensile strength at a printing speed of 50 mm/s at 230 °C, Tough PLA specimens at 230 °C with a printing speed of 25 mm/s, and PLA/Tough PLA hybrid specimens at 220 °C with a printing speed of 50 mm/s. When the tensile strength values of the specimens produced using 25 and 50 mm/s printing speed are analysed, it can be said that the highest tensile strength value for each of the hybrid PLA/Tough PLA, PLA, and Tough PLA specimens is reached as a result of the production at 230 °C. It was determined that the Young’s modulus values given in Table 2 are similar to the tensile strength values; they behave in the same way in cases where the tensile strength values decrease and increase.

### 3.2. Three-Point Bending Tests

**Figure 7:**
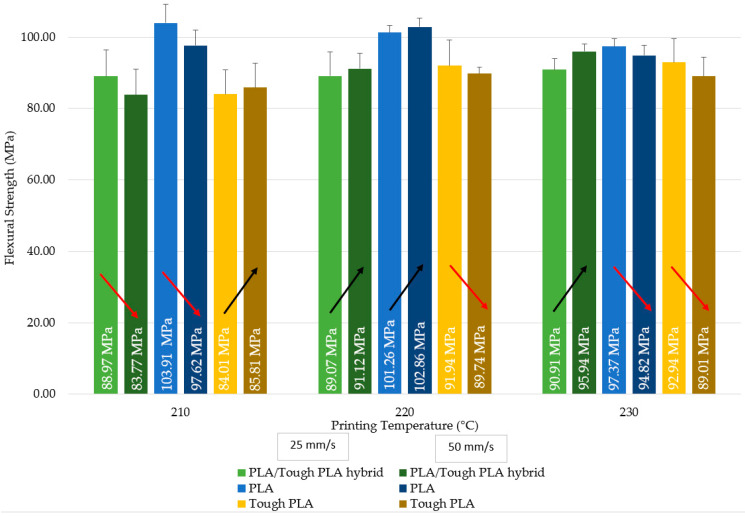
Flexural strength values comparison of the PLA/Tough PLA hybrid, PLA, and Tough PLA specimens. (Arrows indicate the increase and decrease in the flexural strength values of the relevant materials by comparison).

The three-point bending test results of PLA/Tough PLA hybrid material specimens are given in Figure 7 and Table 3. According to Figure 7, the highest flexural strength value for the specimens produced at 210 °C is obtained by the specimens produced with 25 mm/s printing speed, while the highest flexural strength value among the specimens produced at 220 and 230 °C is obtained by the specimens produced with 50 mm/s printing speed. In terms of all production conditions, the highest flexural strength value is 95.94 MPa for the specimens produced at 230 °C with a printing speed of 50 mm/s, and the lowest flexural strength value is 83.97 MPa for the specimens produced at 210 °C with a printing speed of 50 mm/s. No significant increase or decrease is observed in the flexural strength value of the specimens produced with 25 mm/s printing speed as the printing temperature increases; however, an increase in the flexural strength values of the specimens produced with 50 mm/s printing speed is observed as the printing temperature increases. The specimens produced at 230 °C have the highest flexural strength value for both printing speeds. The specimens produced at 230 °C have the highest flexural modulus values in accordance with the flexural strength results.

When Figure 7 is evaluated, the highest flexural strength value for the PLA specimens produced at 210 and 230 °C is obtained by the specimens produced at 25 mm/s printing speed, while the highest flexural strength value among the specimens produced at 220 °C is obtained by the specimens produced at 50 mm/s printing speed. In terms of all production conditions, the highest flexural strength value is 103.91 MPa for the specimens produced at 210 °C with a printing speed of 25 mm/s, and the lowest flexural strength value is 94.82 MPa for the specimens produced at 230 °C with a printing speed of 50 mm/s. When examining the printing temperature, the flexural strength value of the specimens produced with a printing speed of 25 mm/s decreases as the printing temperature increases. When an evaluation is made in terms of printing speed, the specimens produced with 25 mm/s printing speed reach the highest flexural strength value at 210 °C, while the highest value at 50 mm/s printing speed is reached in the specimens produced at 220 °C. When an evaluation is made in terms of flexural modulus, it is revealed that the specimens produced at 210 °C have the highest flexural modulus values.

The highest flexural strength value for the Tough PLA specimens produced at 220 and 230 °C is obtained by the specimens produced with 25 mm/s, while the highest flexural strength value among the specimens produced at 210 °C is obtained by the specimens produced with 50 mm/s. In terms of all production conditions, the highest flexural strength value is 92.94 MPa for the specimens produced at 230 °C with 25 mm/s, and the lowest flexural strength value is 84.01 MPa for the specimens produced at 210 °C with 25 mm/s. The flexural strength value of the specimens produced with 25 mm/s increases as the printing temperature increases. The highest flexural strength value of the specimens produced with 25 mm/s is reached at 230 °C, while the highest value at 50 mm/s printing speed is reached in the specimens produced at 220 °C. The specimens produced at 220 °C have the highest flexural modulus.

It is observed that PLA specimens have the highest flexural strength value for 25 mm/s printing speed. PLA specimens have the highest flexural strength value for the specimens produced at 210 and 220 °C with 50 mm/s, and PLA/Tough PLA hybrid specimens for 230 °C. When the optimum production conditions are evaluated in terms of the production parameters taken as basis in this study, PLA specimens reach the highest value in terms of flexural strength at 210 °C at 25 mm/s, Tough PLA specimens at 230 °C with 25 mm/s, and PLA/Tough PLA hybrid specimens at 230 °C with 50 mm/s.

When the flexural strength results of PLA/Tough PLA hybrid, PLA, and Tough PLA specimens produced with 25 mm/s are evaluated, it is observed that the flexural strength of PLA/Tough PLA hybrid and Tough PLA specimens increase with the increase in printing temperature, while the flexural strength of PLA specimens decreases with the increase in printing temperature. For the specimens produced with 50 mm/s, it is determined that the flexural strengths of PLA/Tough PLA hybrid and Tough PLA specimens increase with the increase in printing temperature. For PLA/Tough PLA hybrid material, it is found that the printing temperature at which the highest values are reached for both printing speeds in terms of flexural strength was 230 °C. The flexural strength values of the hybrid PLA/Tough PLA specimens produced at 230 °C with 50 mm/s are higher than the flexural strength values of both PLA and Tough PLA specimens. Likewise, it is found that the highest flexural strength values for Tough PLA specimens according to both printing speeds are obtained as a result of production at 230 °C. The characteristic performance of samples produced using 3D printing technology depends on the properties of the filament material, as well as on the strength and stability of the bonds formed between the layers of the sample, which are usually determined by the 3D printing parameters [24]. Printing temperature, one of the primary and most effective production parameters of the FDM technique, affects the rheological properties, crystallinity, deformation, thermal, and mechanical properties of the sections formed by polymeric materials. FDM production parameters cause the mechanical properties to change by affecting the bond strength between layers and printing fibre lines; in addition, the printing temperature parameter affects the FDM printability and macromechanical properties of the printed part [25,26,27]. Printing at low temperatures causes the molten polymer to have low fluidity and high viscosity. It results in low bonding ability between the layers and the printing lines of the printed geometry. As the printing temperature increases, the viscosity decreases, and therefore the polymer fusion and fusion between the layers increase. This paves the way for better results in mechanical properties [19,28,29]. If the printing temperature is too high, the fluidity of the extruded material is very high, almost liquid, and the material may even be thermally degraded. During such a printing, complete solidification does not occur both between the printing lines and between the layers. Therefore, both the dimensional accuracy of the part and the mechanical properties are affected [18,30,31,32]. Low production speed prolongs the production time. Therefore, residual thermal stress occurs between layers and between printing lines due to heating and cooling cycles. Dimensional deviations occur in the part as a result of residual thermal stress [33].

### 3.3. Absorptive Tests

Typical diffusion properties of PLA/Tough PLA hybrid, PLA, and Tough PLA specimens presented as the percentage weight increase during moisture absorption are given in Table 4 as a function of exposure time. Each experimental point represents the average of five measurements made on individual specimens.

It is seen that the weight measurement results of the specimens after the determined periods are close to each other and show similar behaviours with the increase in exposure time. According to these results, it is noticed that the specimens exposed to distilled water for three days, one week, and two weeks gain weight in the range of 0.5–1.5%, and all specimens exhibit similar values as a result of the same periodic distilled water exposure. Thanks to the porous structure arising from the nature of the production with the FDM technique, water uptake can be provided towards the inner layers of the specimens [30,34]. It is found that the specimens absorbed water in three-day, one-week, and two-week exposure periods. It is determined that the water absorption ability reaches equilibrium after one week of exposure and exhibits similar values after two weeks of exposure. Ecker et al. [35] investigated the mechanical properties and water absorption behaviour of PLA and PLA/wood composites prepared by FDM technique and injection moulding. They reported that the specimens produced by the FDM technique reached an equilibrium level after 140–160 h of distilled water exposure.

### 3.4. Morphological Analyses

**Figure 8:**
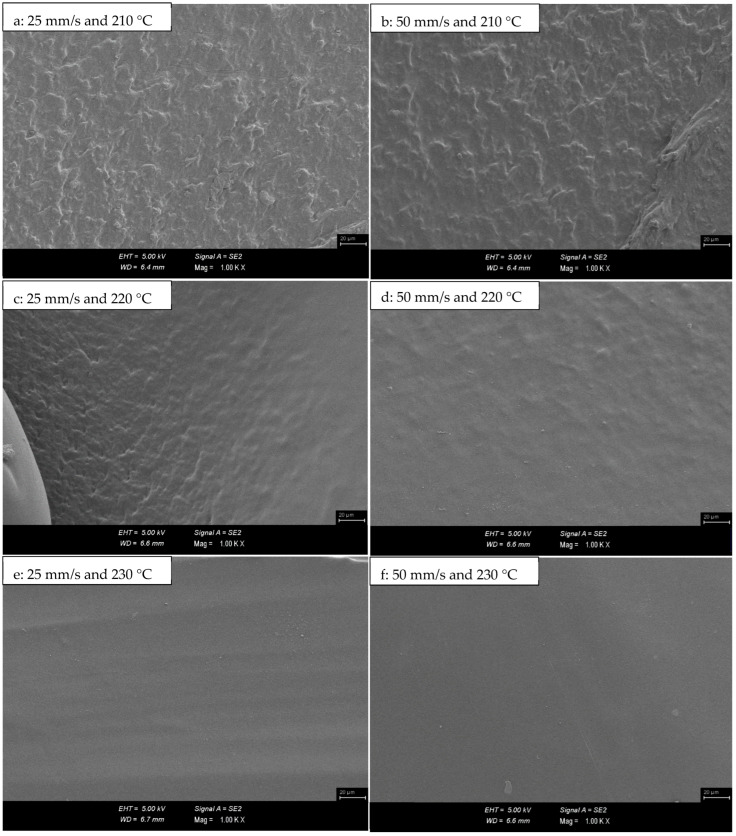
SEM micrographs of PLA/Tough PLA hybrid material specimens.

**Figure 9:**
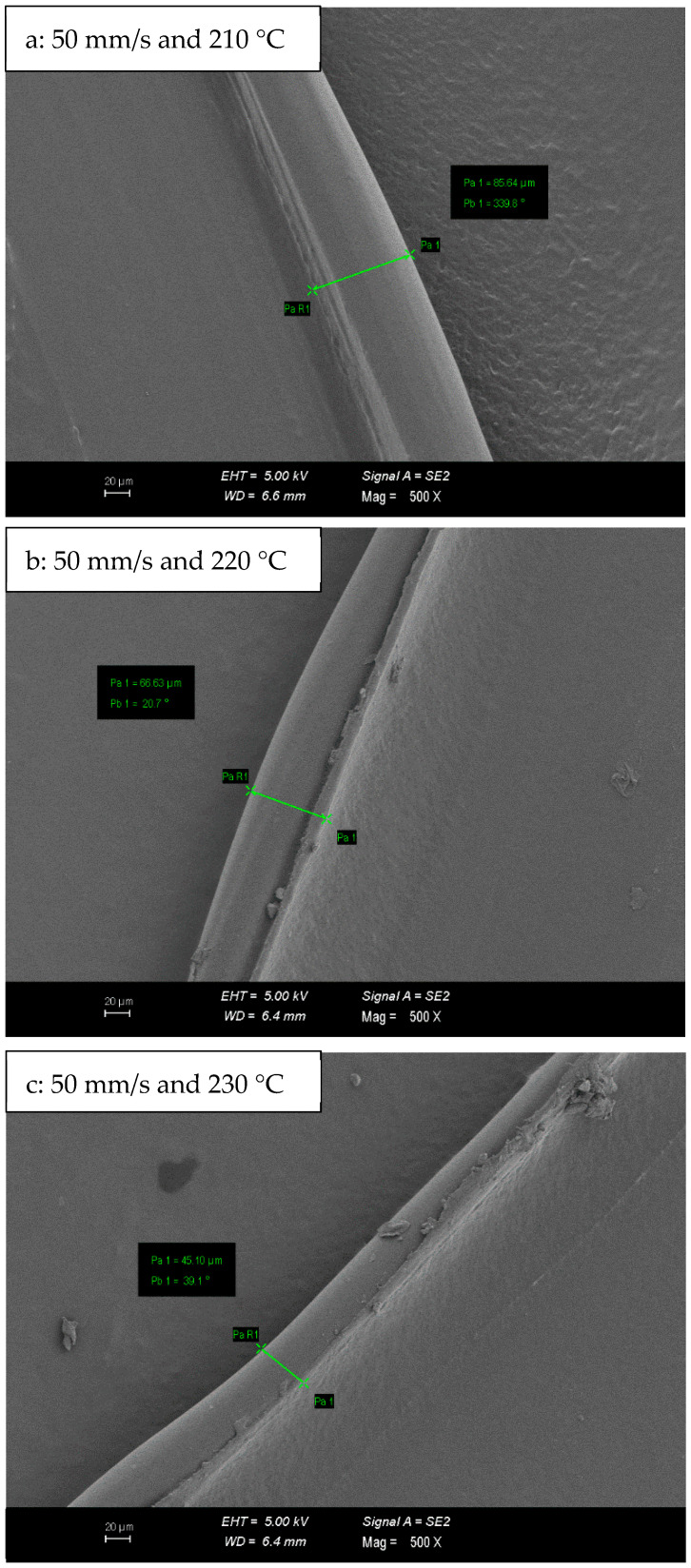
Print fibre wall width measurements of PLA/Tough PLA hybrid material specimens.

SEM micrographs of PLA/Tough PLA hybrid material specimens are given in Figure 8. In the micrographs given in Figure 8, the bumpy and rough ground morphology, which is most prominent in Figure 8a–c, is replaced by a smooth and flat ground morphology in Figure 8e,f. Concerning mechanical properties, the specimens produced at 230 °C have higher mechanical properties. Likewise, it is observed in the micrographs in Figure 9 that the print fibre wall widths of PLA/Tough PLA hybrid specimens produced with 50 mm/s at 210, 220, and 230 °C printing temperature decrease in prominence with increasing printing temperature. In FDM technology, when production with high printing temperature is preferred, a high fluidity level and interfacial bonding ability emerge, which causes the specimens to exhibit higher mechanical properties [35,36,37]. Due to the high printing temperature, the porous structure forms between the layers, and between the printing fibres in the same layer, it is relatively better filled by the filament material, and therefore a smoother surface morphology is formed.

## 4. Conclusions

In this study, a hybrid material was produced using PLA and Tough PLA materials using a 3D printer with FDM technology, which was revised and made suitable for hybrid composite material production. The characterisation properties of PLA/Tough PLA hybrid, PLA, and Tough PLA materials were investigated mechanically using tensile and three-point bending tests, absorptively, and morphologically by SEM analysis. The results obtained accordingly are itemised below.

It is observed that the flexural strength values of PLA/Tough PLA hybrid and Tough PLA specimens increase with the increase in printing temperature. The highest flexural strength value (95.94 MPa) of PLA/Tough PLA specimens is observed in the ones printed with 25 mm/s at 230 °C. This value is measured as 92.94 MPa for Tough PLA specimens and occurs in those printed with 25 mm/s at 230 °C.

PLA specimens reach the highest value in terms of tensile strength with a printing speed of 50 mm/s at 230 °C, Tough PLA specimens with a speed of 25 mm/s at 230 °C, and PLA/Tough PLA hybrid specimens with a speed of 50 mm/s at 220 °C. These values are 38.56 MPa, 26.25 MPa, and 37.40 MPa, respectively.

The weight measurement results of the specimens after the determined periods are close to each other and show similar behaviours with the increase in exposure time. Specimens absorb water in three-day, one-week, and two-week exposure periods. It is determined that the water absorption ability reaches the equilibrium level after one week of exposure and exhibits similar values after two weeks of exposure.

The bumpy and rough ground morphology formed on the surface of the samples produced at 210 °C and 220 °C is replaced by a smooth and flat ground morphology with the increase in production temperature.

## Figures and Tables

**Table 1:** Printing parameters of this study.

Product Parameters	Unit	Value
Moulding technology	-	FDM
Materials	-	PLA, Tough PLA
Volumetric hybridisation ratio	(%)	50/50
Filament diameter	(mm)	1.75
Nozzle diameter	(mm)	0.4
Nozzle temperature	(°C)	210, 220, 230
Bed temperature	(°C)	50
Printing accuracy	(mm)	±0.1
Printing speed	(mm/s)	25, 50
Layer height	(mm)	0.2
Infill density	(%)	100
Raster angle	(°)	45
Environmental temperature	(°C)	28
Environmental humidity	(%)	45 (±5%)

**Table 2:** Young’s modulus values of tensile test specimens.

		Young’s Modulus (GPa)					
		PLA/Tough PLA Hybrid		PLA		Tough PLA	
	Printing Speed (mm/s)	25	50	25	50	25	50
Printing Temperature (°C)	210	2.58 (±0.27)	2.56 (±0.27)	3.04 (±0.08)	3.32 (±0.14)	2.35 (±0.24)	2.33 (±0.08)
	220	2.81 (±0.47)	3.00 (±0.20)	2.55 (±0.61)	2.95 (±0.16)	2.10 (±0.07)	2.28 (±0.13)
	230	2.92 (±0.20)	2.80 (±0.13)	2.85 (±0.27)	2.79 (±0.13)	2.54 (±0.15)	2.52 (±0.14)

**Table 3:** Flexural modulus values of three-point bending test specimens.

		Flexural Modulus (GPa)					
		PLA/Tough PLA Hybrid		PLA		Tough PLA	
	Printing Speed (mm/s)	25	50	25	50	25	50
Printing Temperature (°C)	210	2.00 (±0.17)	1.92 (±0.17)	2.44 (±0.12)	2.64 (±0.19)	1.77 (±0.12)	1.80 (±0.21)
	220	2.05 (±0.18)	1.93 (±0.09)	2.32 (±0.09)	2.44 (±0.12)	1.88 (±0.10)	1.89 (±0.19)
	230	2.09 (±0.09)	2.25 (±0.10)	2.23 (±0.16)	2.45 (±0.12)	1.76 (±0.11)	1.86 (±0.15)

**Table 4:** Absorptive test result of the PLA/Tough PLA hybrid, PLA, and Tough PLA specimens. (Arrows indicate the increase and decrease of the absorptive test results values of the relevant rows by comparison).

			Increase in Weight (%)					
			PLA/Tough PLA Hybrid		PLA		Tough PLA	
	Printing Speed (mm/s)		25	50	25	50	25	50
Printing Temperature (°C)	210	3 days 1 week 2 weeks	0.51 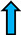 1.49 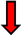 1.19	0.53 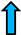 1.38 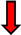 1.23	0.50 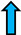 1.55 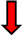 1.39	0.60 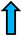 1.50 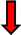 1.30	0.51 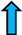 1.51 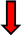 1.41	0.52 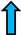 1.57 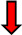 1.33
	220	3 days 1 week 2 weeks	0.58 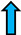 1.50 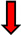 1.28	0.55 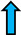 1.47 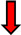 1.25	0.57 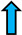 1.47 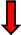 1.35	0.58 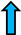 1.61 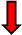 1.39	0.62 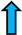 1.47 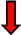 1.38	0.59 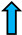 1.48 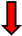 1.25
	230	3 days 1 week 2 weeks	0.53 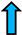 1.43 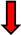 1.29	0.59 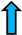 1.48 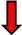 1.30	0.70 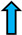 1.49 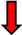 1.41	0.56 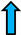 1.50 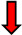 1.49	0.60 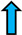 1.44 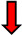 1.48	0.62 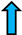 1.45 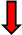 1.38

## Data Availability

The original contributions presented in the study are included in the article, further inquiries can be directed to the corresponding author.
